# Cell heterogeneity, rather than the cell storage solution, affects the behavior of mesenchymal stem cells in vitro and in vivo

**DOI:** 10.1186/s13287-021-02450-2

**Published:** 2021-07-13

**Authors:** Yong-Hong Wang, Ya-Chao Tao, Dong-Bo Wu, Meng-Lan Wang, Hong Tang, En-Qiang Chen

**Affiliations:** 1 Center of Infectious Diseases, West China Hospital, Sichuan University, Chengdu, 610041 People’s Republic of China; 2 Division of Infectious Diseases, State Key Laboratory of Biotherapy, Sichuan University, Chengdu, Sichuan 610041 People’s Republic of China

**Keywords:** Heterogeneity, Mesenchymal stem cells, Storage solution, Morphology, Function

## Abstract

**Background:**

Mesenchymal stem cells (MSCs) have to be expanded in vitro to reach a sufficient cell dose for the treatment of various diseases. During the process of expansion, some obstacles remain to be overcome. The purpose of this study was to investigate the effects of storage solutions and heterogeneity on the behavior of MSCs in vitro and in vivo.

**Methods:**

Umbilical cord MSCs (UC-MSCs) of similar sizes within normal ranges were suspended in three different storage solutions, phosphate buffer solution, normal saline, and Dulbecco’s modified Eagle medium. Then, the ultrastructure, viability, and safety of these cells were compared. Other two UC-MSC populations of different sizes were categorized based on their mean diameters. The ultrastructure, proliferation, immunosuppression, hepatic differentiation potential, and number of senescent cells were investigated and compared. The survival rates of mice after the infusion of UC-MSCs of different sizes were compared.

**Results:**

For UC-MSCs suspended in different storage solutions, the cell apoptosis rates, ultrastructure, and survival rates of mice were similar, and no differences were observed. Cells with a diameter of 19.14 ± 4.89 μm were categorized as the larger UC-MSC population, and cells with a diameter of 15.58 ± 3.81 μm were categorized as the smaller population. The mean diameter of the larger UC-MSC population was significantly larger than that of the smaller UC-MSC population (*p* < 0.01). Smaller UC-MSCs had more powerful proliferation and immunosuppressive potential and a higher nucleus-cytoplasm ratio than those of large UC-MSCs. The number of cells positive for β-galactosidase staining was higher in the larger UC-MSC population than in the smaller UC-MSC population. The survival rates of mice receiving 1 × 10^6^ or 2 × 10^6^ smaller UC-MSCs were 100%, both of which were higher than those of mice receiving the same amounts of larger UC-MSCs (*p* < 0.01). The cause of mouse death was explored and it was found that some larger UC-MSCs accumulated in the pulmonary capillary in dead mice.

**Conclusion:**

Different storage solutions showed no significant effects on cell behavior, whereas heterogeneity was quite prevalent in MSC populations and might limit cells application. Hence, it is necessary to establish a more precise standardization for culture-expanded MSCs.

**Supplementary Information:**

The online version contains supplementary material available at 10.1186/s13287-021-02450-2.

## Introduction

Mesenchymal stem cells (MSCs) are multipotent stromal cells with the potential to differentiate into a variety of cell lineages and represent novel promising candidates to overcome clinical challenges. MSCs can be harvested from multiple tissues, including bone marrow, adipose tissue, the skin, menstrual blood, and umbilical cord blood [1]. MSCs have been approved to treat a broad range of diseases, including chondral defects of the knee [2], steroid-resistant graft-versus-host disease [3], and complex perianal fistulas in Crohn’s disease [4].

The quality of the pharmaceutical production of cells should be strictly tested before they are introduced in the clinic. In most cases, MSCs have to be expanded in vitro to reach a sufficient cell dose. Therefore, it is an essential prerequisite to guarantee the unified quality control of cell products during the course of expansion. However, large-scale expansion of MSCs introduces a bias into the culture process that is difficult to control. In the course of long-term research, we found that some MSC populations derived from human umbilical cord were safe for animal experiments, while some were not, and the reason for this difference remains unclear. This is a real-world problem that affects the safety of MSCs and needs to be overcome before the translation of MSCs to the clinic.

Cell storage solutions and MSCs themselves were thought to be possible reasons for the different outcomes of mice in animal experiments. MSCs are usually collected and suspended in three common storage solutions: phosphate buffer solution (PBS), normal saline (NS), and Dulbecco’s modified Eagle medium (DMEM). Whether MSCs suspended in the three different solutions may lead to different cell behaviors and have unexpected harmful effects on mouse survival remains unknown. Meanwhile, during the expanded culture, we found that UC-MSC populations isolated from different donors varied greatly in cell sizes. Heterogeneity among different UC-MSC populations may be another contributor to the different outcomes of mice. Cell heterogeneity mainly refers to the differences in morphology and function among cell populations. It has reported that MSCs may display morphological and functional heterogeneity during in vitro culture expansion [5, 6]. Whether MSCs heterogeneity can account for the different outcomes of mice remains unknown.

To better understand the biology and safety of MSCs, our aim was to investigate the effects of storage solutions (using MSCs of similar sizes within normal ranges) and heterogeneity (using MSCs of different sizes) on the behavior of UC-MSCs in vitro and in vivo, with the hope of providing more information allowing the use of MSCs in the clinic.

## Materials and methods

### Culture and identification of UC-MSCs

The human umbilical cord MSCs (UC-MSCs) used in this study were produced by Hui Rong Tong Chuang Biological Technology Co., Ltd. UC-MSCs were isolated from afterbirth tissue, which were donated voluntarily by consenting mothers. We also obtained approval for using afterbirth tissues by consenting mothers. Briefly, under sterile conditions, the surface membrane, umbilical vein, and artery were removed, and the remaining Wharton’s jelly was washed and cut as small as possible. Then, the tissue was tiled on culture dishes containing DMEM (Gibco, USA), 20% fetal bovine serum (Gibco, USA) and 1% penicillin/streptomycin. The tissue was then placed in a 37 °C, 5% CO_2_ incubator. Approximately 7–10 days later, cell growth was observed. The obtained cells continued to be cultured and were passaged. Cells were harvested and identified at passage 3 according to their morphology, the expression of cellular markers, and their differentiation potential. UC-MSCs from passages 3–6 were used in experiments.

### Morphological observation of UC-MSCs

UC-MSCs were grown to confluence and serially passaged. Adherent cells were observed under an Olympus CKX53 optical microscope (Tokyo, Japan).

UC-MSCs of similar sizes within normal ranges (with a diameter of 15.81 ± 4.12 μm) were collected and resuspended in three common storage solutions (PBS, NS and DMEM). These storage solutions were all refrigerated at 4 °C and recovered to room temperature before use. The morphological changes of UC-MSCs suspended in the three different solutions were visualized under the optical microscope.

During the expanded culture in vitro, we found that UC-MSCs isolated from one donor were larger than those isolated from another donor. We then measured the sizes of MSCs from the two donors at passage 4 and categorized them as larger and smaller UC-MSC populations based on their mean diameters.

### Expression of specific markers on UC-MSCs

The phenotype profile of UC-MSCs was assessed by flow cytometry analysis (BD Accuri^TM^ C6 flow cytometer) using PE-labeled CD34, CD90, CD45, and CD105 antibodies. The PE-labeled IgG1 was used as the isotype control. Harvested cells were washed twice with PBS, and then resuspended in PBS. Approximately 100 μL of the suspension was treated with conjugated antibodies against CD34, CD45, CD90, and CD105 at dilutions recommended by the manufacturer. All the antibodies were purchased from BD.

### Differentiation potential of UC-MSCs

Induction of adipogenic differentiation was performed using an adipogenic differentiation medium kit (Cyagen Biosciences Inc., China) as previously reported [7]. UC-MSCs were seeded in six-well plates and treated with adipogenic medium for 21 days, with the medium changed 3 times per week. Adipogenesis was assessed by Oil Red O staining.

To induce osteogenic differentiation, UC-MSCs were seeded in gelatin-coated six-well plates and treated with osteogenic medium (Cyagen Biosciences Inc., China) for 21 days, with the medium changed 3 times per week as previously described [8]. Osteogenesis was assessed by alizarin red staining. All photos were taken under an Olympus CKX53 microscope.

### Apoptosis of UC-MSCs suspended in different storage solutions

Apoptosis was detected using an Annexin V- Fluorescein isothiocyanate (FITC) apoptosis detection kit (BD Biosciences, USA) according to the manufacturer’s protocol. Collected UC-MSCs were washed twice with PBS, suspended in 500 μL 1× binding buffer, and stained with 5 μL of annexin V-FITC conjugate and 10 μL of propidium iodide (PI) solution. After incubation for 15 min in the dark at room temperature, stained cells were analyzed by flow cytometry (BD Accuri^TM^ C6 flow cytometer).

### Ultrastructural analysis of UC-MSCs

Ultrastructural analysis of UC-MSCs was performed using transmission electron microscopy (TEM). UC-MSCs of different sizes were collected and immersed in 2.5% glutaraldehyde for 48 h at 4 °C, followed by fixation with 1% osmium tetroxide for 30 min at 4 °C. The specimens were dehydrated using a graded ethanol series (30, 50, 70, 80, 90, and 100%) for approximately 15 min at each step and were then incubated in pure acetone for 20 min. Subsequently, the specimens were embedded in epoxy resin at 60 °C for 24 h. Ultrathin sections were obtained using an ultramicrotome and were then stained with uranyl acetate and lead citrate. The specimens were examined under a JEOL-JEM 1010 microscope (Tokyo, Japan). The nucleus-cytoplasm ratio was calculated as the area of the nucleus/(AREAC-AREANUC), where AREAC is the area of the cell and AREANUC is the area of the nucleus, as previously reported [9].

### Viability and proliferation of different-sized UC-MSCs

Cell viability and proliferation were measured according to the protocol of the 3-(4,5-dimethyl-2-thiazolyl)-2, 5-diphenyl-2-H-tetrazolium bromide (MTT) Cell Proliferation and Cytotoxicity Assay Kit (Beyotime Biotechnology, China). UC-MSCs at passage 4 were seeded in 96-well plates and incubated for 2, 3, 4, and 5 days. MTT was added to each well, and UC-MSCs were incubated at 37 °C in the dark for 4 h. MTT was then removed, and 150 μL of DMSO was added to each well. Absorbance at 570 nm was measured using a Model ELX800 microplate reader (Bio-Tek Instruments). All experiments were performed in triplicate.

### Real-time PCR analysis

Total RNA was isolated from UC-MSCs using TRIzol Reagent (Invitrogen, USA). Quantitative real-time (qRT)-PCR was performed using a LightCycler FastStart DNA MasterPLUS SYBR Green I kit (Roche). GAPDH was used as an endogenous control. The primers (Table 1) specific to target genes were synthesized by TSINGKE Biological Technology (Beijing, China). Template cDNA was added to the reaction mixture, and amplification was initiated with a 10 min template denaturation step at 95 °C, followed by 40 cycles of 95 °C for 15 s and 60 °C for 1 min. All samples were amplified in triplicate.

**Table 1:** Primers used in this study

Gene	Forward (5′-3′)	Reverse (5′-3′)
*GAPDH*	GGAGCGAGATCCCTCCAAAAT	GGCTGTTGTCATACTTCTCATGG
*IDO*	TGCCAACTCTCCAAGAAAC	GCAGTCTCCATCACGAAAT
*HGF*	GCTATCGGGGTAAAGACCTACA	CGTAGCGTACCTCTGGATTGC
*VEGF*	CTGGGCTGTTCTCGCTT	CCCCTCTCCTCTTCCTTCT
*TGF-β*	CTAATGGTGGAAACCCACAACG	TATCGCCAGGAATTGTTGCTG
*IL-6*	CCTGAACCTTCCAAAGATGGC	TTCACCAGGCAAGTCTCCTCA
*IL-10*	GACTTTAAGGGTTACCTGGGTTG	TCACATGCGCCTTGATGTCTG

### Animal experiments

Animal experiments were conducted with approval from the Ethical Committee for Animal Experimentation of Sichuan University. Male C57BL/6 mice, 6–8 weeks old, were maintained in a controlled environment (24 °C, 55% humidity and 12 h day/night rhythm) and had free access to food and water. UC-MSCs (1 × 10^6^) with a mean diameter within the normal ranges were collected, suspended in the three different solutions, and injected into mice through the tail vein as soon as possible (4 mice/group). The survival rates of the mice were monitored for 4 h after administration of UC-MSCs.

Approximately 1 × 10^6^ and 2 × 10^6^ larger and smaller UC-MSCs suspended in PBS were intravenously injected into mice through the tail vein (4 mice/group), respectively. The survival rates of mice that received UC-MSCs of different sizes were monitored for 4 h after administration.

### Hepatic differentiation of UC-MSCs

A three-step differentiation protocol using a Hepatogenic Differentiation kit (Cyagen Biosciences Inc., China) was used to induce hepatic differentiation of UC-MSCs according to the manufacturer’s protocol. Hepatic induction was performed over a period of 3 weeks. Briefly, in the first differentiation step, after reaching 80% confluence, UC-MSCs were treated with Hepatogenic Differentiation Basal Medium A supplemented with epidermal growth factor (EGF) and basal fibroblast growth factor (bFGF) and cultured for 2 days. Then, cells were incubated with 10 mL Hepatogenic Differentiation Basal Medium B supplemented with 2 μL hepatocyte growth factor (HGF), 1 μL bFGF, and 10 μL nicotinamide during the hepatic induction stage. The cell medium was changed every 3 days for 7 days. In the last step, the hepatocyte mature stage, cells were cultured with 10 mL Hepatogenic Differentiation Basal Medium C supplemented with 2 μL oncostatin M, 5 μL dexamethasone, and 100 μL ITS+Premix. The cell medium was changed every 3 days for 7–14 days. After 7 to 14 days of maturation, cells were collected for various evaluation tests.

### Functional evaluation and comparison of hepatocyte-like cells (HLCs)

Indocyanine green (ICG, Sigma) was added to the culture at a final concentration of 1 mg/mL. Cells were incubated at 37 °C for 1 h and then washed three times with PBS. ICG uptake was visualized under a light microscope.

Glycogen storage of HLCs derived from the size-based UC-MSC populations was detected using a periodic acid-Schiff (PAS) kit (Solarbio, China) according to the manufacturer’s instructions. Briefly, cells were fixed in 4% formaldehyde for 30 min, oxidized in 1% periodic acid for 10 min, and rinsed twice with water. Subsequently, cells were treated with Schiff’s reagent for 15 min and then rinsed with water. Glycogen storage was assessed under a light microscope (Olympus, Tokyo, Japan). ImageJ software (National Institutes of Health, MD, USA) was used to quantify the area of positive cells by binarizing images followed by area extraction.

### Detection of senescence

Cell senescence was determined using a β-galactosidase (β-gal) staining kit (Beyotime, China). UC-MSCs were fixed and stained in the solution at 37 °C for 24 h. Then, cells positive for β-gal activity were observed under a microscope.

### Immunofluorescence

UC-MSCs of different sizes were transfected with GFP (WZ Bioscience, Inc., China), and these cells were harvested and then administered to mice through the tail vein. Tissue samples (from the liver, lung and heart) obtained from mice were collected and fixed in 4% paraformaldehyde overnight, cryopreserved in 30% sucrose overnight, frozen in OCT compound (Thermo Scientific), and stored at − 80 °C. The cryopreserved samples were cut into 8 μm sections. The slides were washed with PBS and permeabilized with 0.2% Triton X-100 for 20 min. The slides were then washed three times with PBS and incubated with 10% goat serum in PBS for 1 h at room temperature. Next, the slides were incubated with the primary CoraLite®594- conjugated CD31 antibody (Proteintech, China) in a humidified chamber at 4 °C overnight. Nuclei were stained with DAPI (Life Technologies, USA) for 10 min. The slides were washed three times with PBS and mounted with ProLong^TM^ Gold Antifade Mountant (Invitrogen, USA). Images were taken under a Nikon Eclipse Ti epifluorescence microscope.

### Statistics analysis

Independent experiments were repeated three times. Statistical analysis was performed using SPSS 20.0. All quantitative data are presented as the mean ± standard deviation (SD). Non-parametric test was used to analyze the differences between groups, and the results were considered statistically significant if *p* < 0.05.

## Results

### Identification of UC-MSCs

**Fig. 1:**
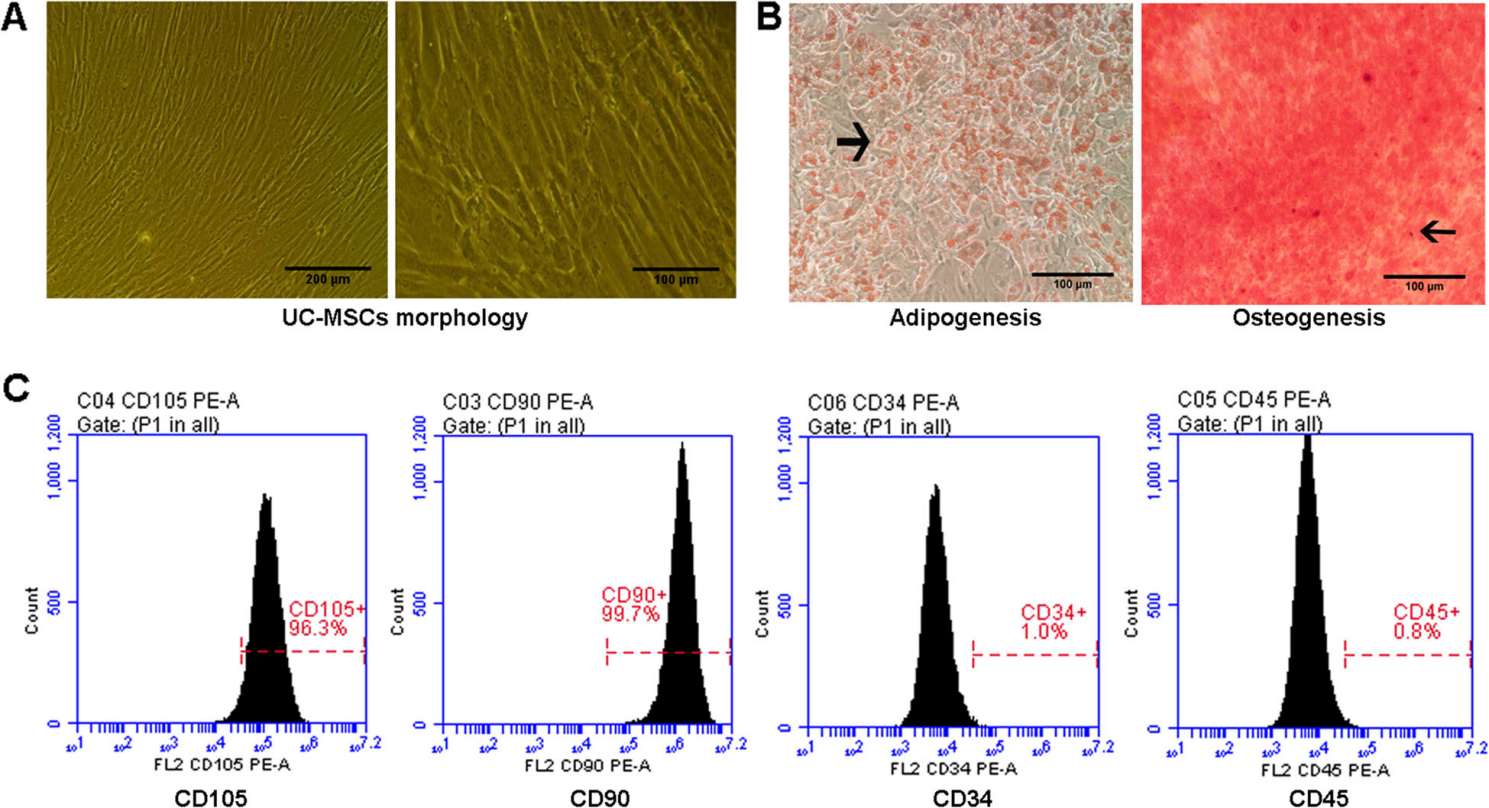
Identification of UC-MSCs. **A** Observation of cell morphology under an optical microscope. Left: scale bar = 200 μm, right: scale bar = 100 μm. **B** Detection of adipogenesis and osteogenesis capacity of cells. Differentiation results are indicated with arrow. Scale bar = 100 μm. **C** Analysis of cell surface markers by flow cytometry

UC-MSCs cultured in vitro were adherent to the plastic tissue culture dishes and had a spindle-like morphology (Fig. 1A). Under standard cell induction conditions, the adipogenic and osteogenic potential of UC-MSCs were evaluated. Oil Red O-positive lipid droplets and alizarin red-positive calcium deposits were observed after induction differentiation for 21 days (Fig. 1B).

Cell surface markers were analyzed by flow cytometry. The results showed that cells were positive for CD105 (96.3%) and CD90 (99.7%) and negative for CD34 (1.0%) and CD45 (0.8%) (Fig. 1C), demonstrating that the cultured cells expressed characteristic stem cell-associated surface markers.

### Effects of different storage solutions on the vitality and function of UC-MSCs with similar sizes in normal ranges

**Fig. 2:**
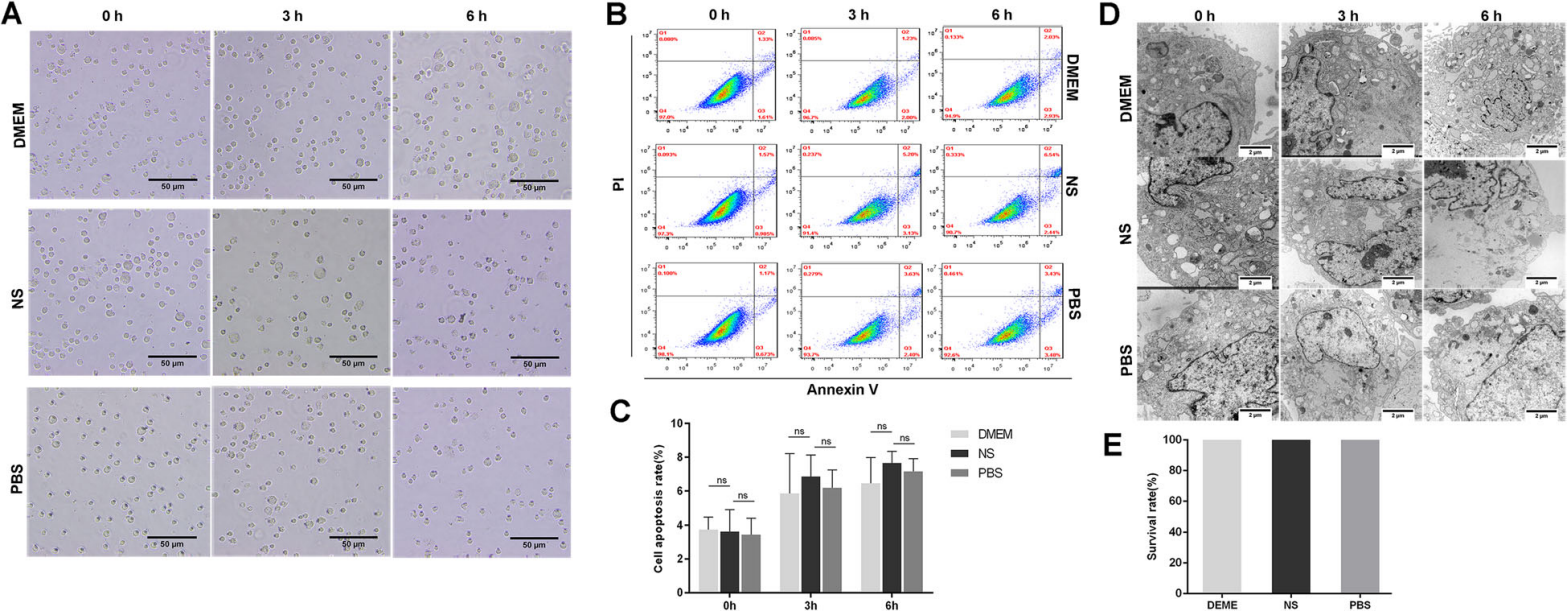
Effects of different storage solutions on the behavior of UC-MSCs. **A** Morphology of UC-MSCs suspended in DMEM, NS, and PBS over time. Scale bar = 50 μm. **B**, **C** Calculation of the apoptosis rates of UC-MSCs suspended in DMEM, NS, and PBS over time. **D** Ultrastructure analysis of UC-MSCs suspended in the three different storage solutions. Scale bar = 2 μm. **E** Comparison of the survival rates of mice (n = 4 for each group) injected with UC-MSCs suspended in the three different storage solutions. Data are presented as the mean ± SD of three independent experiments. ns, not significant

UC-MSCs suspended in DMEM, NS, and PBS showed a similar round appearance (Fig. 2A). The apoptosis rates of UC-MSCs in DMEM, NS, and PBS were calculated to be approximately 3.64% ± 0.62%, 3.62% ± 1.27%, and 3.44% ± 0.97% at 0 h, 5.85% ± 1.28%, 6.85% ± 0.74%, and 6.19% ± 0.61% at 3 h, 6.45% ± 0.88%, 7.66% ± 0.39%, and 7.15% ± 0.44% at 6 h, respectively. No significant differences were observed (Fig. 2B, C).

The effects of storage solutions on the ultrastructure of UC-MSCs were further investigated using TEM. The main organelles were similar among the UC-MSCs in the different solutions at the same time point, whereas all the UC-MSCs suspended in the different storage media swelled over time, accompanied by the swelling of cytoplasm and the dissolution of some organelles (Fig. 2D).

Approximately 1 × 10^6^ UC-MSCs suspended in the three different solutions were injected into mice, and the survival rates of mice were monitored and compared. No mice died after administration of UC-MSCs suspended in the different storage solutions (Fig. 2E). Overall, it seemed that the storage solutions had little effect on the nature of UC-MSCs.

### Effects of size-based heterogeneity on the vitality and behavior of UC-MSCs

**Fig. 3:**
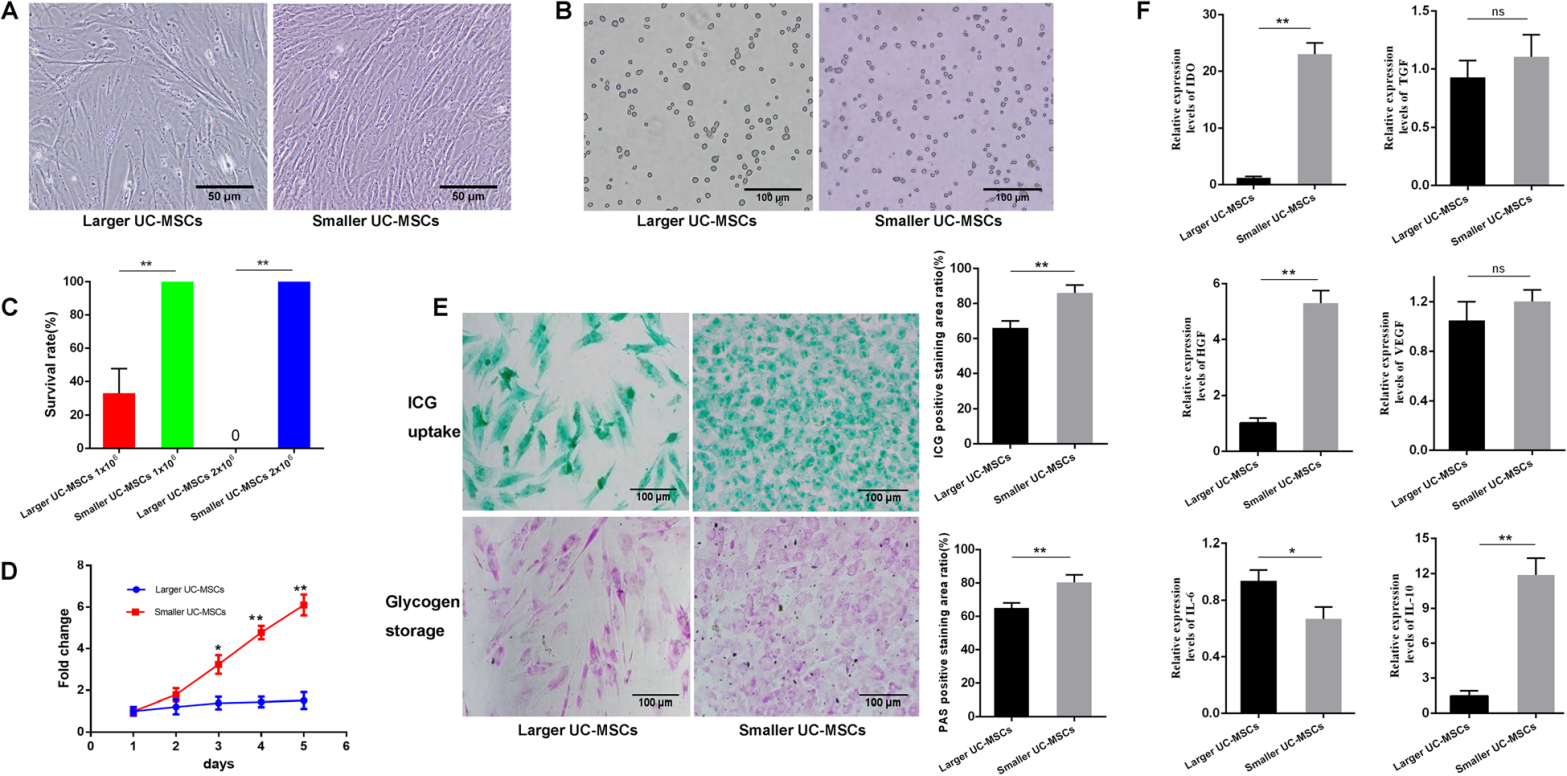
Effects of morphology on the characteristics of UC-MSCs. **A** Morphology of UC-MSCs of different sizes when they were attached to the culture dishes. Scale bar = 50 μm. **B** Morphology of UC-MSCs of different sizes when they were suspended in PBS. Scale bar = 100 μm. **C** Comparison of the survival rates of mice (n = 4 for each group) injected with UC-MSCs of different sizes. **D** Comparison of the proliferation potential of the two different sized UC-MSC populations. **E** Functional comparison of HLCs derived from UC-MSCs of the larger and smallerpopulations. Positive staining was quantified using ImageJ software, and the results were compared between the two populations. **F** Comparsion of the gene expression levels of cytokines (IDO, HGF, VEGF, TGF-β, IL-6, and IL-10) involved in immunity between larger and smaller UC-MSCs after stimulation with IFN-γ. All data are presented as the mean ± SD of three independent experiments. *p < 0.05, **p < 0.01; ns, not significant.

UC-MSCs were spindle-like when adhering to the culture dishes (Fig. 3A) and showed a round or an oval shape when suspended in PBS (Fig. 3B). We took pictures of suspended UC-MSCs under an optical microscope and measured the longest diameters of cells one by one in the pictures using ImageJ software. Cells with a diameter of 15.58 ± 3.81 μm were categorized as the smaller UC-MSC population, and cells with a diameter of 19.14 ± 4.89 μm were categorized as the larger population. The mean diameter of the larger UC-MSC population was significantly larger than that of the smaller population (*p* < 0.05). Approximately 1 × 10^6^ and 2 × 10^6^ UC-MSCs of different sizes were intravenously injected into mice, and the survival rates of mice were monitored and compared. The survival rates of mice receiving 1 × 10^6^ or 2 × 10^6^ smaller UC-MSCs were both 100%, higher than those of mice receiving the same amounts of larger UC-MSCs (*p* < 0.01, Fig. 3C).

Further study showed that smaller UC-MSCs showed a more potent proliferation potential than that of larger UC-MSCs according to the MTT assay (*p* < 0.01, Fig. 3D). Assays were performed to investigate the function of HLCs derived from UC-MSCs of different sizes. ICG uptake stained as green deposits, and glycogen storage stained as light pink/purple deposits. More positive staining of ICG and glycogen granules was observed in the cytoplasm of HLCs derived from the smaller UC-MSC population. The ratio of positive staining was significantly higher in the smaller UC-MSC population than in the larger population (*p* < 0.01, Fig. 3E). These results indicate that UC-MSCs can successfully differentiate into HLCs, and the synthetic and storage functions of HLCs derived from the smaller UC-MSC population may be more powerful than those derived from the larger UC-MSC population.

To compare the immunosuppressive function of UC-MSCs of different sizes, we detected the levels of some cytokines involved in immunity in UC-MSCs stimulated with interferon-γ (IFN-γ, 10 ng/mL) using qRT-PCR. The expression levels of indoleamine 2,3-dioxygenase (IDO), transforming growth factor beta (TGF-β), vascular endothelial growth factor (VEGF), hepatocyte growth factor (HGF), interleukin 6 (IL-6), and interleukin 10 (IL-10) were measured and compared. As shown in Fig. 3F, compared to those in the larger UC-MSC population, the transcript levels of IDO (*p* < 0.01), HGF (*p* < 0.01), and IL-10 (p < 0.01) were significantly higher in the smaller UC-MSC population stimulated with IFN-γ, and the level of IL-6 (*p* < 0.05) was lower, indicating that smaller UC-MSCs may have a more powerful immunosuppressive ability than larger UC-MSCs.

**Fig. 4:**
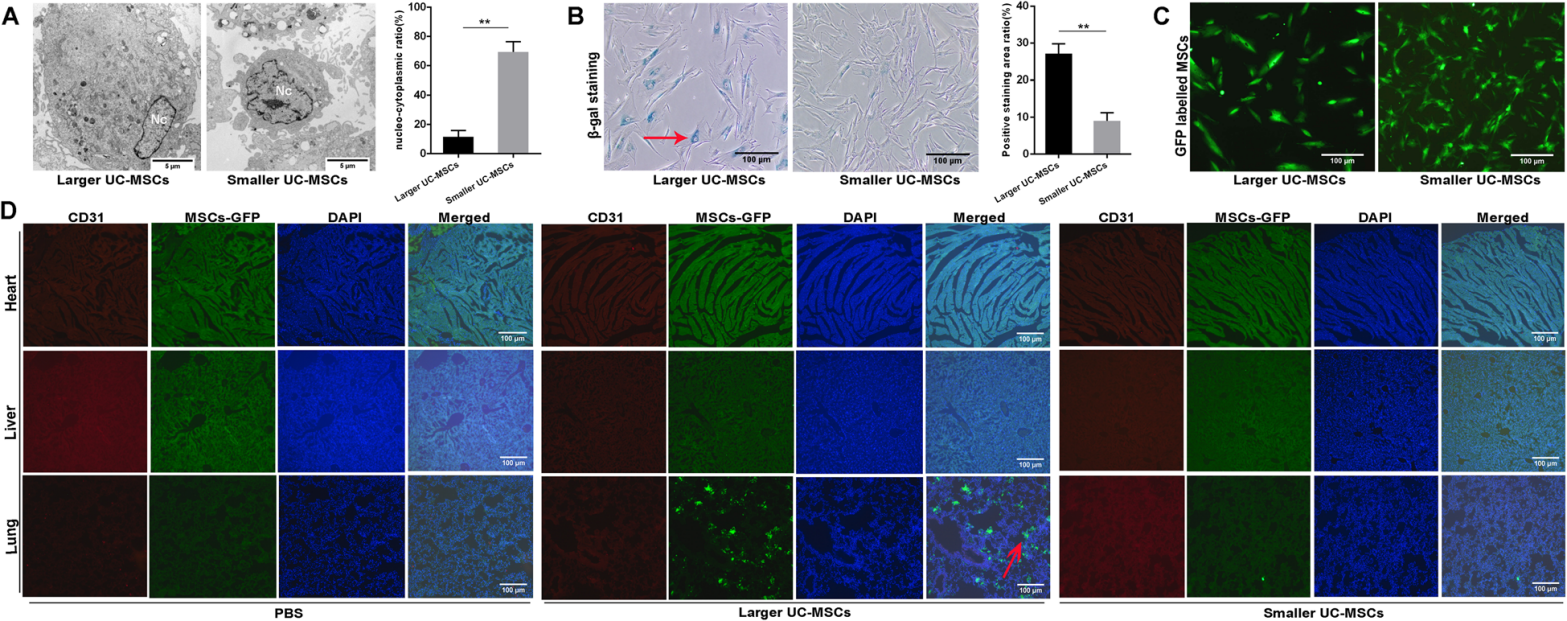
Effects of size-based morphological differences on the behavior of UC-MSCs. **A** Ultrastructure analysis of UC-MSCs of the larger and smaller populations. In addition, the nucleus to cytoplasm ratio was calculated and compared. Nc, nucleus. Scale bar = 5 μm. **B** Comparison of the number of senescent cells (red arrow) in the populations of UC-MSCs of different sizes. In addition, the positive results were quantified and compared. Scale bar = 100 μm. **C** Observation of UC-MSCs labeled with GFP. Scale bar = 100 μm. **D** Immunofluorescence analysis of the heart, liver, and lung from mice after infusion of UC-MSCs of different sizes. Scale bar = 100 μm. All data are presented as the mean ± SD. **p < 0.01

Via TEM, we observed the difference of the ultrastructure of larger and smaller UC-MSCs. Smaller UC-MSCs had a higher nucleus-cytoplasm ratio than larger UC-MSCs (*p* < 0.01, Fig. 4A).

Senescence was detected by the β-gal assay. The results showed that the number of cells positive for β-gal staining was higher in the larger UC-MSC population than in the smaller UC-MSC population (*p* < 0.01, Fig. 4B). Positive results for β-gal are indicators of senescent cells.

### Accumulation of larger UC-MSCs in lung tissue

UC-MSCs of different sizes were labeled with GFP (Fig. 4C). The cause of mouse death after injection of larger UC-MSCs was investigated. Heart, liver, and lung tissues were harvested and assessed by immunofluorescence analysis (Fig. 4D). The results revealed that larger UC-MSCs labeled with GFP accumulated in the pulmonary capillary lumen (red arrow), which might have been one the causes of mouse death.

## Discussion

Although great success has been achieved in pre-clinical and clinical trials of MSCs, some obstacles remain to be overcome, such as the storage solutions used and cell heterogeneity, which may affect the properties and behaviors of cells. In the present study, we verified that UC-MSCs shared certain common properties, including a fibroblast-like morphology, surface maker expression, and differentiation capacity, in vitro. UC-MSCs of similar sizes within normal ranges showed a comparable morphology, mice survival rates, and ultrastructure when suspended in three different storage solutions (DMEM, NS, PBS), indicating that these common storage solutions had little effect on the behavior of MSCs.

Cell heterogeneity may be another contributing factor to the different survival rates of mice. Our study defined cells as larger and smaller UC-MSC populations based on the mean cell diameter and revealed size-based morphological differences between the two UC-MSC populations, although the UC-MSCs were all isolated from the human umbilical cord. Morphological heterogeneity may ultimately affect cell function, as a previous study reported that the chondrogenic differentiation capacity of UC-MSCs was strongly correlated with morphological data based on size and shape features [10]. In the present study, we also found that the smaller UC-MSC population showed more powerful immunosuppression and hepatic differentiation potential than those of the larger UC-MSC population.

The proliferative rate of MSCs may be delayed within 2–3 months during the expanded culture, and they may ultimately enter the senescent state [11]. In our study, more cells positive for senescence-associated β-gal were observed in the larger UC-MSC population, and these larger cells were less proliferative. The asynchronous cell senescence between the two UC-MSC populations may drive different cell activities and behaviors. In addition, compared to that of the larger UC-MSC population, the smaller UC-MSC population displayed a higher nucleus-cytoplasm ratio. A high nucleus-cytoplasm ratio may be, to some extent, associated with the proliferative potential of cells, as it has been found in stem cells at an early stage isolated from menstrual blood and liver tissue [12, 13]. Another study also suggested that the larger UC-MSCs may represent a collection of cells that no longer undergo the normal cell cycle, whereas smaller cells are mitotically active [14]. Collectively, smaller UC-MSCs seemed to be more “naïve” and more active in mitosis, whereas larger cells might be restricted in terms of their proliferation, immunosuppression, and hepatic differentiation potential.

In the present study, we observed that some mice died immediately after intravenous infusion of larger UC-MSCs, and the cause of mouse death was explored. In our previous study, intravenously delivered UC-MSCs first entered the lung under physiological conditions and were trapped there even 4 h later (see the supplement). Thus, we inferred that pulmonary embolism could be one of the main possible causes of mouse death. Subsequent larger MSCs accumulation in pulmonary capillaries supported our inference. Other preclinical and clinical studies have also reported pulmonary embolisms after MSCs intravenous transplantation [15, 16]. However, some existing evidences have shown cerebral embolisms after intracarotid transplantation in the shock model [17, 18]. The reason for the different sites of embolism may be related to the transplantation route. The findings in our study revealed that larger MSCs might be more likely to induce embolism. Selecting smaller MSCs using cell strainer will reduce the risk of embolism. However, cell strainer may be not suitable for mass MSCs screening, and equipment for large-scale cell selection has to be developed. More importantly, figuring out the reasons for the generation of different sized MSCs will fundamentally help to improve the heterogeneity among MSC populations and reduce the incidence of embolism.

When MSCs are suspended in storage solution, more cell clumps may be formed over time [19]. However, in our study, all the UC-MSCs were administered to mice as soon as possible after harvesting. Thus, the nature of MSCs themselves likely leads to the different outcomes in animal experiments, namely, the morphological and functional heterogeneity of UC-MSCs may affect cell behavior [10, 20]. Hence, considering the morphological and functional heterogeneity of MSCs, it is necessary to screen for optimal MSCs to guarantee their safety and efficacy in the clinic.

It has been proven that freeze-thawing impairs cell survival and function [19, 21]. Meanwhile, more senescent cells, which are characterized by enlarged morphology, decreased expression of surface markers, and declined differentiation potential, may be observed with increasing passage number [11, 14]. However, all the UC-MSCs used in the present study were off-the-shelf (cryopreserved products), and these thawed MSCs were cultured under the same standard conditions and collected at the same cell passage for study. Hence, we speculate that the heterogeneity between the two UC-MSC populations may be attributed to the host source, namely, donor-to-donor variation [22]. Age, obesity, and state of health may affect the functions of MSCs [23–27]. The neonate who generated larger UC-MSCs was a boy weighing 3.15 kg whose mother was a 28-year-old healthy primipara. And the neonate who generated smaller UC-MSCs was a boy weighing 3.4 kg, and his mother was a 25-year-old healthy primipara. Both mothers and babies were free from infections of cytomegalovirus, hepatitis B virus, hepatitis C virus, human immunodeficiency virus (HIV), and syphilis. Their routine examinations and hepatic and renal functions were all normal. Besides, the two mothers had no history of smoking and drinking. We speculated that the difference between the ages of the two mothers and the difference between the weighs of the two babies had a limited effect on the heterogeneity of UC-MSCs. Instead, the genetic backgrounds of donors may account for the heterogeneity of UC-MSCs in our study, based on the previous study [28].

Although we found heterogeneous UC-MSCs isolated from two donors both in vitro and in vivo, we did not explore the transcriptome difference of the two UC-MSC populations. Another study using single-cell RNA sequencing analysis revealed highly variable genes (HVGs) expressed in UC-MSCs isolated from three donors, and these HVGs were associated with the functional characteristics of classic MSCs [29], which helps to illustrate the underlying molecular mechanism of heterogeneity. However, Huang et al. [30] reported limited heterogeneity of UC-MSCs, regardless of donor and passage, and the reason for the different results might be the existence of confounding factors in the latter study, such as batch and cell cycle effects.

Heterogeneity is also found in cells obtained from a single donor [31]. It is generally believed that cell clusters derived from a single cell should be functionally homogeneous stem cells, but this is not always the case. Single-cell colonies are not necessarily homogeneous subpopulations, and colonies cannot accurately represent the entire putative stem cell subpopulation [32]. In the present study, mild morphological differences within a UC-MSC population derived from a single donor were observed. Cell-to-cell variation among MSCs within a single population can become evident during culture expansion, and culture-expanded MSCs are actually a mixture of cell subpopulations [33, 34]. Heterogeneity is so pervasive that it is reasonable to infer that MSC populations are intrinsically heterogeneous [35]. Therefore, current characterization of MSCs, mainly including plastic adherence, differentiation capacity in vitro, and a minimalistic panel of special surface markers, are far from adequate for defining MSCs [36], because they cannot account for cell heterogeneity among MSC populations, as these standards are incomplete in terms of cell morphology and function, nor can they accurately predict cell functions in vivo, as the functions of MSCs in vitro and in vivo may be different.

The reasons for the heterogeneity within a typically expanded UC-MSC population are complex. One reason may be that UC-MSCs are composed of multi-cell-derived cells. These multi-cell-derived cells are initially different both in terms of both gene and protein expressions, leading to heterogenetic progeny. The accumulation of defects and mutations during long-term culture may be other reasons accounting for cell heterogeneity [11]. Cell-to-cell contact may contribute to changes in cell size and morphology [37], but it may not be a fate-determining factor, as Haack-Sorensen et al. [38] reported that cell density had no significant influence on the phenotype of MSCs. Moreover, current manufacturing and culturing protocols may not be conducive to maintaining MSCs homogeneity. Regardless of the reason, measures have to be taken to improve isolation, processing, and culture expansion technologies to reduce cell heterogeneity and ensure the consistency of cell quality. Some cell companies are trying to develop equipment for large-scale culture. With this large-scale cultivation equipment, all operations will be carried out on the machine to guarantee the stability of MSCs quality in the future.

There were some limitations in this study. First, the limited small size of two MSC populations (one of each side) may limit the generalizability of our results; thus, more cell populations will be required to verify the conclusions. Second, we investigated the heterogeneity of MSCs in normal mice, not in pathological mice, and had not directly compared the therapeutic effects between MSCs of different sizes. Third, whether lung diseases, for instance, pulmonary arterial hypertension, affect cell entrapment and embolism in the lung needs further investigation. Moreover, the reasons for the heterogeneity of MSC populations isolated from different donors have to be explored in future to better maintain the homogeneity of MSCs.

## Conclusion

Taken together, our results show that different storage solutions have no significant effects on the behavior of UC-MSCs, whereas heterogeneity is quite prevalent in UC-MSC populations and may limit the application of these cells. However, this heterogeneity can be easily overlooked. The findings of the present study may lay a foundation to better understand MSCs heterogeneity, emphasizing the need to establish a more precise standardization for culture-expanded MSCs.

## Supplementary Information

**Additional file 1.**

## Data Availability

All data generated or analyzed during this study are included in this published article.

## Abbreviations

UC-MSCs: Umbilical cord mesenchymal stem cells
PBS: Phosphate buffer solution
NS: Normal saline
DMEM: Dulbecco’s modified Eagle Medium
ICG: Indocyanine green
PAS: Periodic acid-Schiff
PI: Propidium iodide
EGF: Epidermal growth factor
B-FGF: Basal fibroblast growth factor
HGF: Hepatocyte growth factor
TEM: Transmission electron microscopy
β-gal: β-galactosidase
IDO: Indoleamine 2,3-dioxygenase
VEGF: Vascular endothelial growth factor
TGF-β: Transforming growth factor beta
IL-6: Interleukin 6
IL-10: Interleukin 10
