# Supplementary material for: Cell heterogeneity, rather than the cell storage solution, affects the behavior of mesenchymal stem cells in vitro and in vivo
Source: Stem Cell Res Ther. 2021 Jul 13;12:391. doi: 10.1186/s13287-021-02450-2 (PMC8278752; doi:10.1186/s13287-021-02450-2)
Supplement: Supplementary file 1 — Additional file 1. [file 13287_2021_2450_MOESM1_ESM.docx]

**Supplement**

**
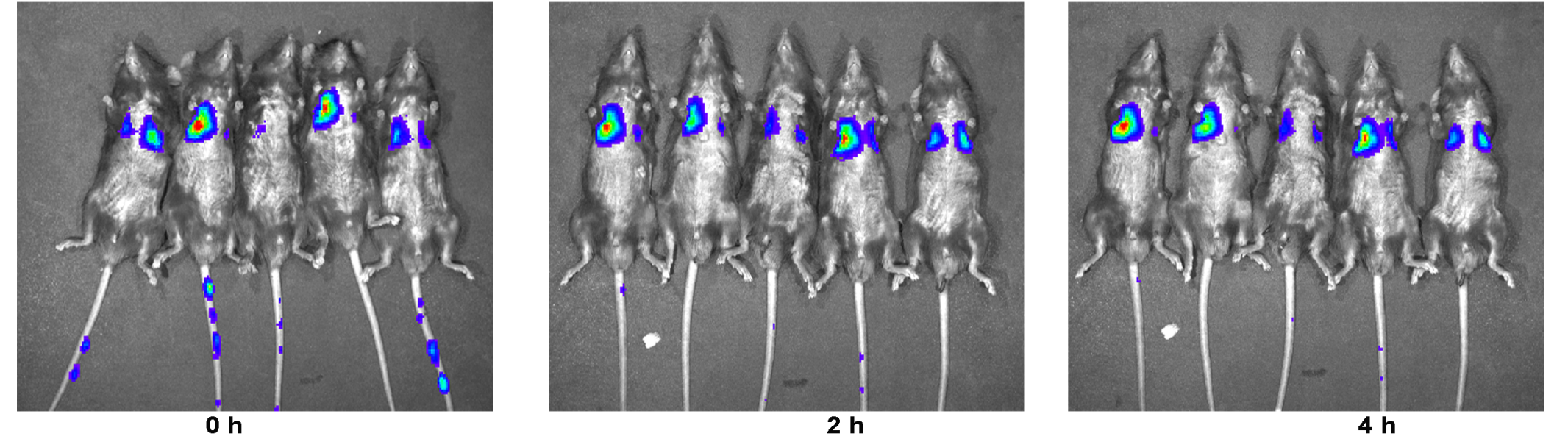
**

Supplement. Observation of UC-MSCs transplanted via tail vein at 0 h, 2 h and 4 h using in vivo bioluminescence imaging.
